# Phytochemical Composition and Antibacterial Activity of *Barleria albostellata* C.B. Clarke Leaf and Stem Extracts

**DOI:** 10.3390/plants12132396

**Published:** 2023-06-21

**Authors:** Serisha Gangaram, Yougasphree Naidoo, Yaser Hassan Dewir, Moganavelli Singh, Johnson Lin, Hosakatte Niranjana Murthy

**Affiliations:** 1 School of Life Sciences, Westville Campus, University of KwaZulu-Natal, Private Bag X54001, Durban 4000, South Africa; 2 Plant Production Department, College of Food and Agriculture Sciences, King Saud University, P.O. Box 2460, Riyadh 11451, Saudi Arabia; 3 Department of Horticultural Science, Chungbuk National University, Cheongju 28644, Republic of Korea

**Keywords:** antibacterial activity, gas chromatography-mass spectrometry, phytochemical analysis

## Abstract

*Barleria albostellata* (Acanthaceae) is a shrub located in South Africa and is relatively understudied. However, plants within this genus are well known for their medicinal and ethnopharmacological properties. This study aimed to characterise the phytochemical compounds and antibacterial efficacies of *B*. *albostellata*. Phytochemical analysis, fluorescence microscopy and gas chromatography-mass spectrometry (GC-MS) analysis were performed to determine the composition of compounds that may be of medicinal importance. Crude leaf and stem extracts (hexane, chloroform and methanol) were subjected to an antibacterial analysis against several pathogenic microorganisms. The qualitative phytochemical screening of leaf and stem extracts revealed the presence various compounds. Fluorescence microscopy qualitatively assessed the leaf and stem powdered material, which displayed various colours under bright and UV light. GC-MS chromatograms represents 10–108 peaks of various compounds detected in the leaf and stem crude extracts. Major pharmacologically active compounds found in the extracts were alpha-amyrin, flavone, phenol, phytol, phytol acetate, squalene and stigmasterol. Crude extracts positively inhibited Gram-positive and Gram-negative bacteria. Significance was established at *p* < 0.05 for all concentrations and treatments. These results indicate that the leaves and stems of *B. albostellata* are rich in bioactive compounds, which could be a potential source of antibacterial agents for treating various diseases linked to the pathogenic bacteria studied. Future discoveries from this plant could advance the use of indigenous traditional medicine and provide novel drug leads.

## 1. Introduction

The dependence on plants as a source of medicine is prevalent in developing countries, especially where traditional medicine plays an important role in health care [1,2,3]. As defined by the World Health Organisation (WHO), traditional medicine is the knowledge, skill and practices based on the beliefs and experiences in various cultures [4]. The demand for herbal medicines worldwide is rapidly increasing due to their availability, low cost and higher safety margins [5,6,7]. The adverse side-effects of conventional medicine are related to certain pharmacological compounds; therefore, creating different therapies with greater effectiveness and bioavailability, with fewer side-effects, is essential [8,9]. Natural compounds isolated from plants have been assumed to remain an essential part of the exploration for new medicines against human diseases [10,11]. 

Africa is considered the cradle of humankind, comprising a rich cultural and biological diversity and with healing practices varying in regions [5,7,12]. Infectious diseases are a serious problem in Africa [13,14], with one of the leading causes of morbidity and mortality arising from bacterial infections (Gram-positive and Gram-negative bacteria) [15,16,17,18]. However, with the use of herbal medicine, certain bacterial infections have been reported to be under control, while others are resistant [19]. According to the WHO, by 2050, there will be approximately 10 million deaths arising from common diseases such as respiratory and urinary tract infections and drug-resistant pathogens, surpassing the number of deaths resulting from cancer [20,21]. Therefore, it is crucial to find alternative solutions, such as herbal extracts, to overcome future threats in the medical field [22].

Throughout the history of mankind, plant extracts have been used to treat various ailments through herbal preparations [23,24,25,26]. These preparations include concoctions, decoctions, infusions and teas [27,28]. Plants are rich in several naturally occurring phytochemicals such as alkaloids, flavonoids, tannins and terpenoids, which have been found to possess antimicrobial properties. These secondary metabolites are important components of a plant’s natural defence mechanisms and are products of primary metabolism [29,30,31,32,33,34,35,36]. 

In South Africa, about 3000 medicinal plants were reported to be used regularly, and from these plants, 38 indigenous species were commercialised [37,38]. These commercialised plant species are available as processed material in various forms, such as capsules, ointments, tablets or teas [39,40]. The verified record of natural products being used in drug discovery [41,42] has provided compelling evidence for increasing the exploration of nature to identify unique active compounds as promising leads for effective drug development [43,44,45]. There has been significant progress in pharmaceutical industries in search of important plant-based medicinal compounds; however, a significant amount of the plant biodiversity remains unexplored [46,47]. Screening plants for biologically active compounds has resulted in the development of new therapeutic drugs to treat several diseases [48].

*Barleria albostellata* C.B. Clarke (Acanthaceae) is a shrub located in South Africa. This shrub extends from Limpopo, Gauteng and Mpumalanga to KwaZulu-Natal [49]. Although *B*. *albostellata* has no recorded practice in traditional medicine, many species within the genus have been widely used in traditional medicine and were confirmed to contain various compounds possessing biological effects such as analgesic, anti-inflammatory, antileukaemic, antihyperglycemic, antitumour, anti-amoebic, antibiotic and virucidal activities [50,51,52,53,54,55]. Amoo et al. [56] examined the antioxidant potential of the methanolic extracts of the leaves and stems of *B. albostellata* and found the greatest flavonoid content in the stems of *B. albostellata* stems. Additionally, Amoo et al. [57] verified the antibacterial, antifungal and anti-inflammatory activity of *B. albostellata* against *B. subtillis*, *S. aureus* and *E. coli*. Thus, further investigation on *B*. *albostellata* is important as this study will provide baseline information on the potential usage of extracts from this plant. There is a scarcity of scientific data on the phytochemical compounds of the leaf and stem extracts of *B*. *albostellata* and its potential antibacterial activity against human pathogens. This study was therefore carried out to bridge these gaps.

## 2. Results and Discussion

### 2.1. Organoleptic Characteristics and Crude Extract Yield of B. albostellata

Organoleptic evaluation is a conventional, qualitative method whereby an individual uses their sight, smell, taste and touch to document the characteristic features of crude drugs. These assessments may serve as a baseline for preliminary phytochemical and pharmacological screening of a plant [58]. The organoleptic features of *B. albostellata* were evaluated by using sensory organs (Table 1). The following features were noted on both adaxial and abaxial surfaces: The leaves were grey-green in colour but lighter on the lower surface. The stems appeared as ‘yellow-buff’ on the upmost internodes and white/cream below. The odour of the leaves was slightly aromatic, whilst the stems were inodorous. The taste was acrid for both leaves and stems. Both surfaces of the leaves were velvety, whereas the stems were woody and glabrescent. According to Shaheen et al. [59], organoleptic studies are important taxonomic parameters, assisting in the verification of several medicinal plant species. 

The highest percentage yield of the crude extracts from *B. albostellata* was obtained from the methanolic extract of the leaves (16.78%), followed by 9.38% from the methanolic stem extract (Table 2). The lowest percentage yield was observed in the hexane stem extract (1.94%). Overall, this suggests that the percentage yield of phyto-compounds in *B. albostellata* was greater in the leaf crude extract than from the stem. Furthermore, this implies that there may be more polar compounds in the leaf extracts and a smaller amount of non-polar compounds in the stem. Therefore, the yield obtained indicates the polarity of the different solvents is related to the plants’ pharmacological importance [60,61]. Each crude extract (hexane, chloroform and methanol) displayed distinct colours (Table 2). Hexane extracts were oily upon evaporation of the solvent, whereas chloroform and methanol dried to a hard-sticky solid. 

### 2.2. Phytochemical Screening for Major Classes of Compounds in Extracts of B. albostellata Using Qualitative Colour Tests, TLC and Fluorescence Analysis

Major compounds identified in leaf and stem extracts of *B. albostellata* were alkaloids, amino acids, carbohydrates, flavonoids, mucilage and gums, phenols, saponins, terpenoids and sterols (Table 3). Fixed oils and fats were present in leaves and absent in the stem extracts. The intensity of compounds in the leaf extracts was greater in comparison to the stem. These compounds can act as defence mechanisms against various microorganisms, herbivores and insects [62,63,64].

Various phytochemicals have been known to contain diverse activities that may help protect against chronic diseases [65,66]. Amoo et al. [56] reported the presence of phenols, iridoids, gallotannins, flavonoids and condensed tannin in the leaves and stems of *B. albostellata*. These authors also found the total iridoid content to be the highest in the leaves of *B. albostellata*. Important pharmacological alkaloids can be found in iridoids, as this compound is known to be a precursor in the biosynthesis of alkaloids [67,68]. Similar compounds were also found in extracts of other *Barleria* species including *B. acuminata* [69], *B. dintteri* [10], *B. cristata* [70,71,72], *B. longiflora* [73] and *B. prionitis* [74]. 

Metabolites such as alkaloids and terpenoids (Table 3) were reported to contain antimicrobial, anticancer and anti-malarial properties [75,76,77,78,79,80]. Additionally, alkaloids have organic and natural constituents with sedative and analgesic roles [81]. Amino acids, carbohydrates and fixed oils and fats (Table 3) were reported to contain antioxidant properties [82,83,84]. Flavonoids and phenols (Table 3) possessed anti-inflammatory, anti-apoptosis, anti-carcinogen and anti-ageing properties [85,86,87,88]. 

Mucilage and gums (Table 3) are used in the treatment of gastric ulcers, for wound healing and as cytoprotective agents, and also contain antipyretic and antiseptic properties [89,90,91,92]. Saponins present in plant extracts (Table 3) are believed to contain anticancer, antioxidant, antiviral and anti-inflammatory properties [93,94]. Additionally, saponins display several hepatoprotective and antimicrobial activities [95]. Sterols were found to reduce cholesterol levels and contain anti-inflammatory and antioxidant properties (Table 3) [96,97,98,99,100]. 

**Figure 1:**
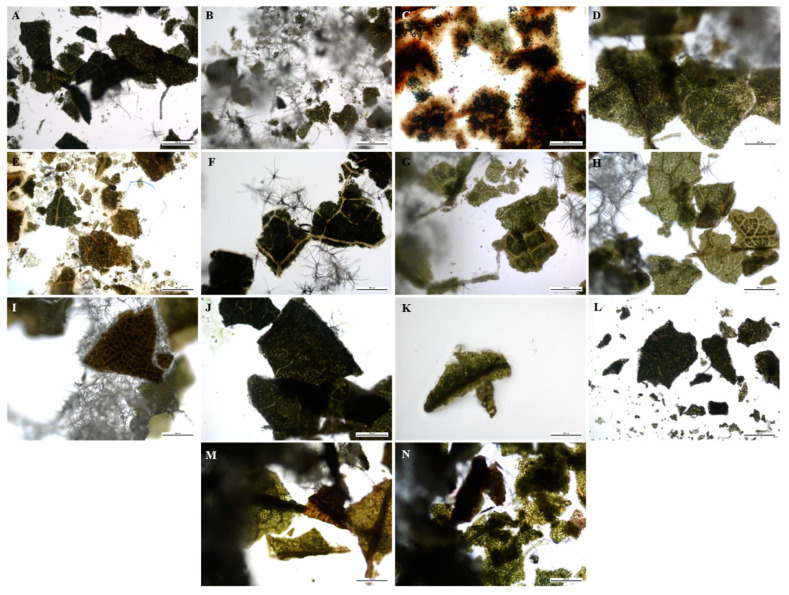
Leaf powder analysis (bright light) of *B. albostellata*. (**A**) Powder; (**B**) powder + water; (**C**) powder + H_2_SO_4_; (**D**) powder + acetic acid; (**E**) powder + aqueous NaOH; (**F**) powder + HCl; (**G**) powder + ethanol; (**H**) powder + ethyl acetate; (**I**) powder + hexane; (**J**) powder + chloroform; (**K**) powder + methanol; (**L**) powder + petroleum ether; (**M**) powder + diethyl ether; (**N**) powder + acetone.

**Figure 2:**
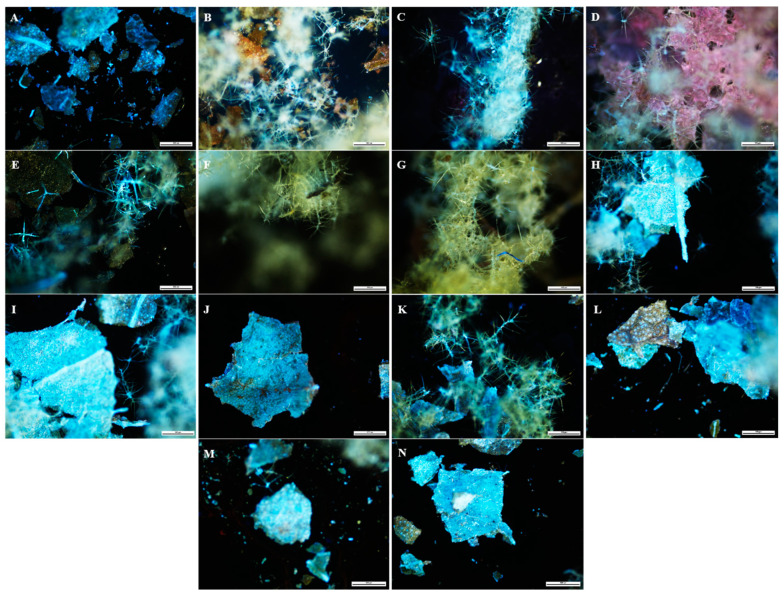
Fluorescence analysis (UV light) of leaf powder from *B. albostellata*. (**A**) Powder; (**B**) powder + water; (**C**) powder + H_2_SO_4_; (**D**) powder + acetic acid; (**E**) powder + aqueous NaOH; (**F**) powder + HCl; (**G**) powder + ethanol; (**H**) powder + ethyl acetate; (**I**) powder + hexane; (**J**) powder + chloroform; (**K**) powder + methanol; (**L**) powder + petroleum ether; (**M**) powder + diethyl ether; (**N**) powder + acetone.

**Figure 3:**
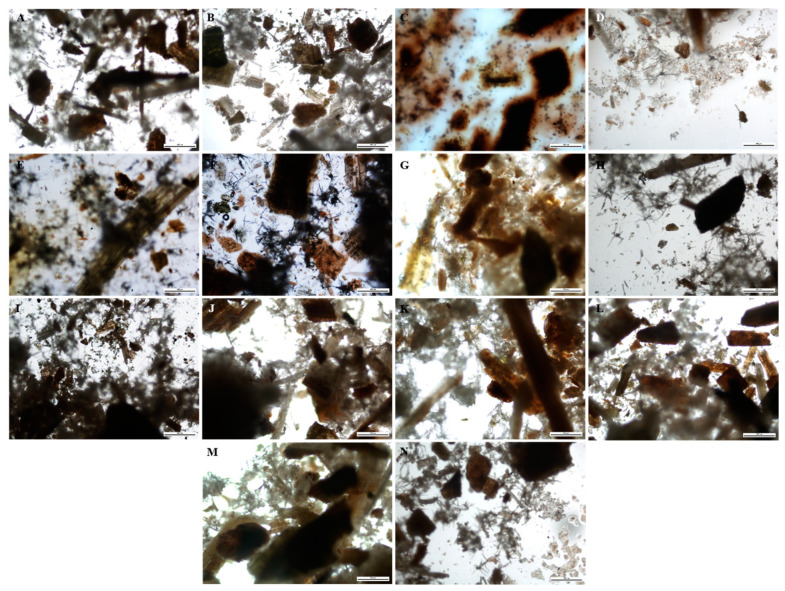
Stem powder analysis (bright light) from *B. albostellata*. (**A**) Powder; (**B**) powder + water; (**C**) powder + H_2_SO_4_; (**D**) powder + acetic acid; (**E**) powder + aqueous NaOH; (**F**) powder + HCl; (**G**) powder + ethanol; (**H**) powder + ethyl acetate; (**I**) powder + hexane; (**J**) powder + chloroform; (**K**) powder + methanol; (**L**) powder + petroleum ether; (**M**) powder + diethyl ether; (**N**) powder + acetone.

**Figure 4:**
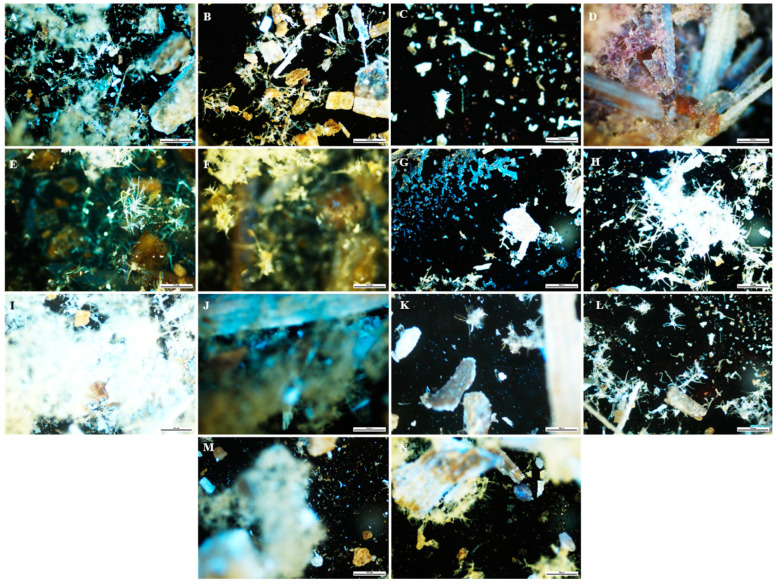
Fluorescence analysis (UV light) of stem powder from *B. albostellata*. (**A**) Powder; (**B**) powder + water; (**C**) powder + H_2_SO_4_; (**D**) powder + acetic acid; (**E**) powder + aqueous NaOH; (**F**) powder + HCl; (**G**) powder + ethanol; (**H**) powder + ethyl acetate; (**I**) powder + hexane; (**J**) powder + chloroform; (**K**) powder + methanol; (**L**) powder + petroleum ether; (**M**) powder + diethyl ether; (**N**) powder + acetone.

A technique used in the qualitative assessment of natural products or crude drugs is fluorescence analysis, which is an important tool for pharmacognostic evaluation [101,102]. The powdered and fluorescence characteristics of the leaf and stem powder of *B*. *albostellata* are presented in Figure 1, Figure 2, Figure 3 and Figure 4. The powdered leaf and stem material treated with several reagents displayed various colours when observed under bright light, and this is compared to the colours observed under UV light. It should be noted that the colours indicated for the powdered leaf and stem material viewed under bright light were described according to the overall appearance. The purity and quality of crude drugs are occasionally authenticated using standard fluorescence characteristics, as certain natural products display no fluorescence in daylight but do so under UV light [103]. Natural products such as berberine alkaloids exhibit fluorescence under UV light but not in daylight [104,105]. As most crude drug materials do not fluoresce, these materials are converted either into fluorescent decomposition- or by-products with the aid of several reagents [101,102,106]. Furthermore, fluorescence analysis can be used to preserve the quality and effectiveness of crude drug materials by easily detecting adulterants and substituents [101,102,107,108]. 

The most prominent UV colour that stands out in both leaf and stem powder was blue (Figure 2 and Figure 4). This colour was observed in multiple plant samples where different reagents were used. According to Chase and Pratt [109], several drugs display duplication of colours, as there are sometimes more than four drugs found in a particular colour group. According to Sridharan and Gounder [110], powdered leaves of *B. montana* were separately exposed to 24 h of light with the addition of various reagents. These included powder + water, + ethanol, + ethyl acetate, + hexane, + chloroform and + acetone. Colours observed from the various regents after 24 h were orange, green, pale and light green, respectively. When these samples were exposed to UV light, colours observed were greenish-orange, light and dark green, pale yellow, yellowish-orange and pale red. Similar fluorescence results listed in this study were detected for certain reagents in the powdered leaf material for *B. noctiflora* [111] and *B. gibsoni* [112].

### 2.3. Phytochemical Screening for Major Classes of Compounds in B. albostellata Extracts Using GC-MS

**Figure 5:**
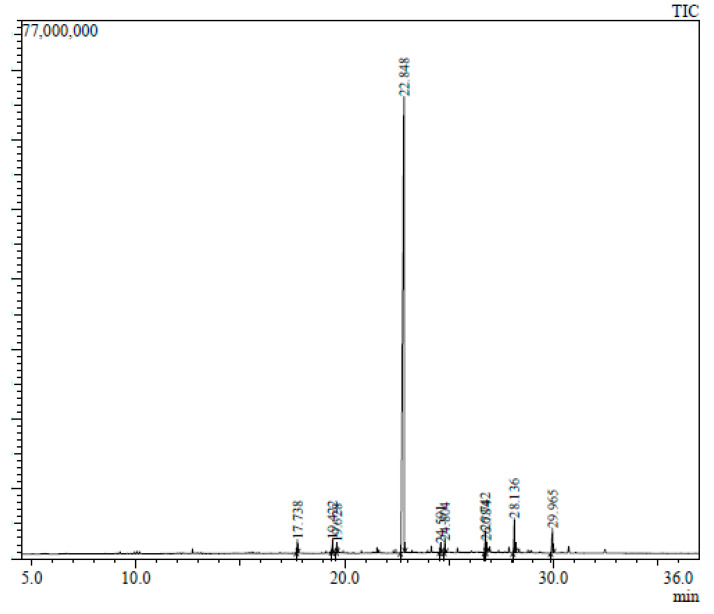
GC-MS chromatogram of leaf hexane extract of *B. albostellata*.

**Figure 6:**
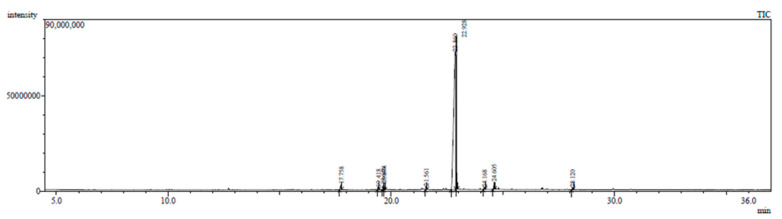
GC-MS chromatogram of leaf chloroform extract of *B. albostellata*.

**Figure 7:**
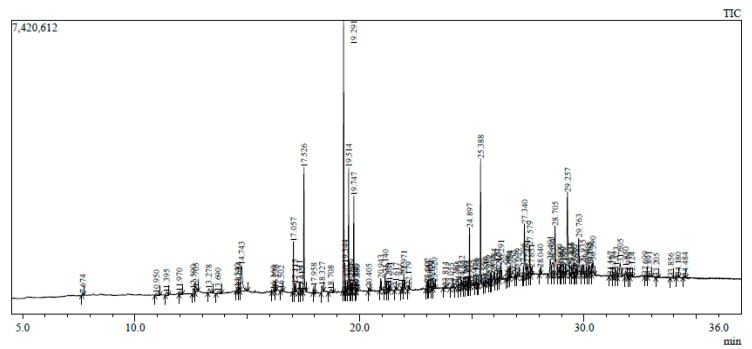
GC-MS chromatogram of leaf methanol extract of *B. albostellata*.

**Figure 8:**
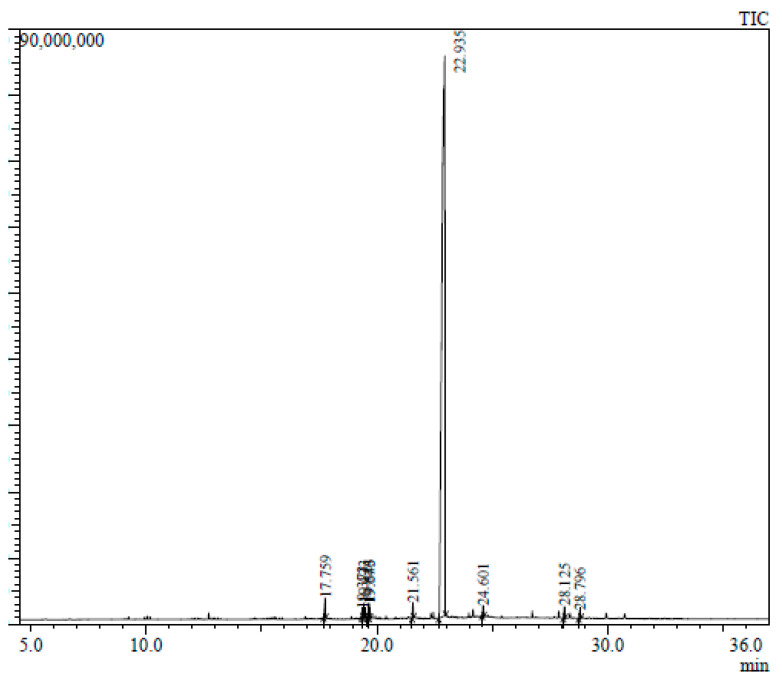
GC-MS chromatogram of stem hexane extract of *B. albostellata*.

**Figure 9:**
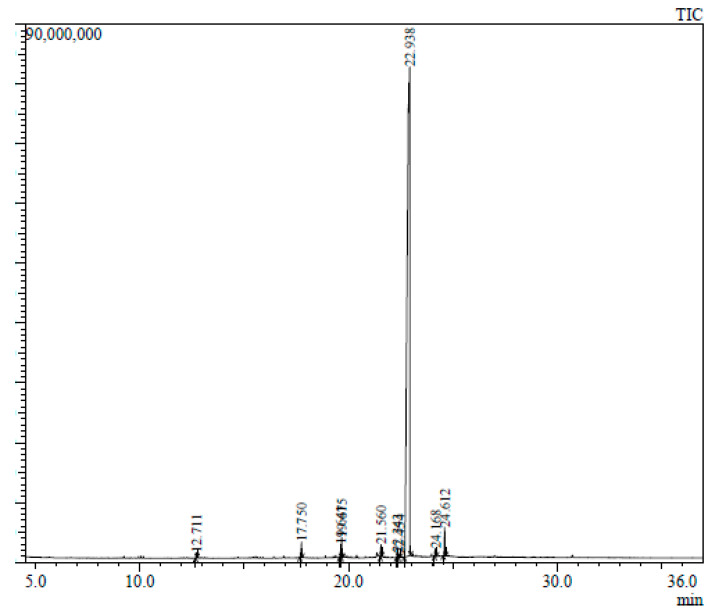
GC-MS chromatogram of stem chloroform extract of *B. albostellata*.

**Figure 10:**
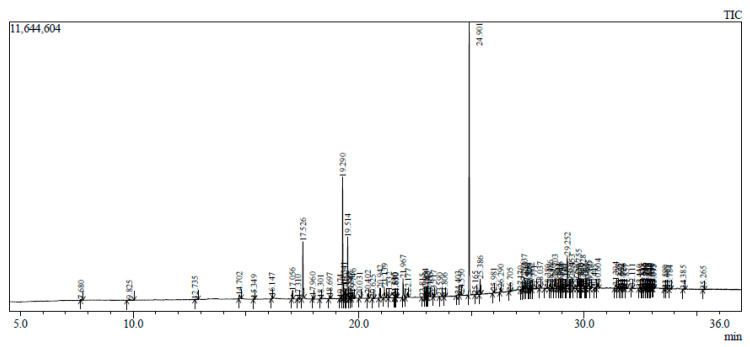
GC-MS chromatogram of stem methanol extract of *B. albostellata*.

The GC-MS chromatogram represents 10 peaks (Figure 5), 10 peaks (Figure 6) and 108 peaks (Figure 7) of various compounds detected in the leaf hexane, chloroform and methanolic extract, respectively. Additionally, 10 peaks (Figure 8), 10 peaks (Figure 9) and 104 peaks (Figure 10) were identified in the stem hexane, chloroform and methanolic extract. This analysis is used for the qualitative and quantitative examination of active compounds in plants [113,114]. Several overlapping peaks were observed in the middle stages of most chromatograms, Figure 5 (19–30 min), Figure 7 (14–33 min), Figure 8 (19–20 min), Figure 9 (19–25 min) and Figure 10 (19–33 min). Each peak in the chromatogram (Figure 5, Figure 6, Figure 7, Figure 8, Figure 9 and Figure 10) represents a signal produced when a compound is washed out with a solvent from the gas chromatography column into the detector [115,116]. 

Numerous small peaks were observed throughout the chromatogram. Major chemical compounds with high peaks and area percentages greater than one were selected and identified in leaf and stem extracts (hexane, chloroform and methanol) (Table 4, Table 5, Table 6, Table 7, Table 8 and Table 9). In certain circumstances, compounds with an area percentage less than one were only mentioned if they played an important role in the genus. The methanolic extracts for both leaf and stem revealed the highest number of compounds. Compounds with an area percentage greater than one were found in the leaf (16) and stem (15) methanolic extracts (Table 6 and Table 9). 

For the leaf hexane chromatogram, the highest peak identified was tetratetracontane, which had the highest percentage area of 3.25% (Table 4). This compound displays antioxidant, cytoprotective and anti-inflammatory activities [113,117]. The lowest peak identified in the leaf hexane (Table 4), stem hexane (Table 7), and stem chloroform (Table 8) chromatogram was pentadecanoic acid, with an area percentage of 1.02%, 1.00% and 1.00%, respectively. It should be noted that pentadecanoic acid has not been reported in any species of *Barleria*; however, this compound is a fatty acid and is found in the milk fat of cows, regulates hormones, improves the immune system and boosts metabolism [118,119].

Octadecanoic acid, 2,3-dihydroxypropyl ester and tetratetracontane displayed the lowest peaks in the leaf chloroform chromatogram, with an area percentage of 1.00% (Table 5). These compounds have not been reported in any species of *Barleria*, though octadecanoic acid, 2,3-dihydroxypropyl ester displays anticancer and antimicrobial activities (Table 10). In the leaf methanolic chromatogram, flavone, 4′,5-dihydroxy-6,7-dimethoxy-exhibited the highest peak, with an area percentage of 11.69% (Table 6). The 13-docosenamide, (Z) displayed the lowest peak, with an average percentage of 2.46%, and has not been reported in any species of *Barleria* (Table 6) but was reported to exhibit antimicrobial properties in *Ludwigia perennis* [120]. 

The highest peak for the stem hexane chromatogram was 4,4,6a,6b,8a,11,11,14b-Octamethyl-1,4,4a,5,6,6a,6b,7,8,8a,9,10,11,12,12a,14,14a,14b-octadecahy-dro-2H-picen-3-one, which had an average percentage of 2.35 (Table 7). This compound was reported to exhibit antibacterial, antioxidant, antitumour and cancer preventives (Table 10). Additionally, the lowest peak on the stem methanol chromatogram was tributyl acetyl citrate (Table 7). Al-Rubaye et al. [121] examined the methanolic leaf extracts of *Sinapis arvensis* for its medicinal properties. These authors found tributyl acetyl citrate to display antioxidant and anti-inflammatory activities. 

The identified compounds illustrated in Table 10, possessed various biological properties of medicinal importance. Several compounds found in the extracts of *B. albostellata* were also noted in other species of *Barleria*. Phyto-compounds such as phenol, 2,4-bis(1,1-dimethylethyl), found in *B. albostellata* (Table 10), were identified in *B. prionitis* [122], *B. montana* [123] and *B. lupulina* [124]. The 9,12,15-octadecatrienoic acid, (Z,Z,Z) (Table 10) was only prominent in *B. buxifolia* [125]. Kumari and Dubey [124] reported on octadecanoic acid (Table 10) in the extracts of *B. lupulina*, while Sriram and Sasikumar [123] found this compound in *B. montana*. Squalene found in *B. albostellata* (Table 10) was also identified in *B. montana* [126], *B. longiflora* [127], *B. courtallica* [128], *B. lupulina* [124] and *B. grandiflora* [129]. 

Eicosane, a solid n-alkane (Table 10), was found in extracts of *B. courtallica* [128], *B. prionitis* [130] and *B. dinteri* [10]. In the extracts of *B. courtallica* [128] and *B. lupulina* [124], phytol and acetate (Table 10) were identified. Furthermore, phytol (Table 10) was reported in *B. montana* [126], *B. longiflora* [127], *B. courtallica* [128], *B. lupulina* [124], *B. strigosa* [131], *B. buxifolia* [125] and *B. prionitis* [130]. Vitamin E (Table 10), a fat-soluble vitamin, was only noted in *B. courtallica* [128]. Flavones, a class of flavonoids, found in the extracts of *B. albostellata* (Table 10) were also reported in *B. prionitis* [132] and *B. acanthoides* [133].

Campesterol found in *B. longiflora* [134]; stigmasterol in *B. courtallica* [128], *B. montana* [123], *B. longiflora* [127], *B. cristata*, *B. prionitis* [135,136] and *B. lupulina* [137]; and beta-sitosterol identified in *B. prionitis* [130], *B. courtallica* [128], *B. montana* [126] and *B. longiflora* [127] are three characteristic phytosterols found in *B. albostellata* (Table 10). Stigmasta-3,5-dien-7-one has only been reported in *B. albostellata* (Table 10), while 13,14-seco-stigmasta-5,14-diene-3*α*-o was noted in *B. prionitis* [138]. Additionally, alpha-amyrin was noted in *B. cristata* [135] and *B. prionitis* [130]. Sujatha et al. [128] and Kumari and Dubey [124] reported the presence of 9,12-octdecadienoic acid (Z,Z) (Table 10) in the extracts of *B. courtallica* and *B. lupulina*, respectively.

To date, 1-heptacosanol; l-(+)-ascorbic acid 2,6-dihexadecanoate; tridecanoic acid; decanedioic acid, dibutyl ester; 1,2,3,5-cyclohexanetetrol; 1,2-15,16-diepoxyhexadecane; 1,4-benzenedicarboxylic acid, bis(2-ethylhexyl) ester; simiarenol; dichloroacetic acid, tridec-2-ynyl ester; 4,4,6a,6b,8a,11,11,14b-octamethyl-1,4,4a,5,6,6a,6b,7,8,8a,9,10,11,12,12a,14,14a,14b-octadecahydro-2H-picen-3-one; alpha amyrenone; acetic acid, 3-hydroxy-6-isopropenyl-4,8a-dimethyl-1,2,3,4,5,6,7,8; and cholest-4-en-3-one, found in *B. albostellata*, were not reported in any species of *Barleria*. Although GC-MS analysis identified the phytochemical constituents present in the hexane, chloroform and methanolic extracts, it should be noted that the most compounds were found in the leaf (Table 6) and stem methanolic (Table 9) extracts. 

**Table 10:** Pharmacological activities of compounds found in *B. albostellata*.

No	Phytochemical Compound	Pharmacological Action	References
1	Pentadecanoic acid	Flavouring agent, lubricants, adhesive agents, ability to regulate hormones, improve the immune system, boost metabolism and inhibit production of uric acid	[118,138,139,140,141]
2	9,12,15-Octadecatrienoic acid, (Z)	Antioxidant, anti-inflammatory, antimicrobial, diuretic, anticancer, antitumour, chemo-preventive properties used in vaccine formulations and reduced complications in COVID-19 patients	[142,143,144,145]
3	Octadecanoic acid	Antimicrobial activity	[146,147,148]
4	13-Docosenamide, (Z)	Antimicrobial activity	[119,149]
5	Squalene	Cosmetics, skin ointments, antioxidant, antitumour, anticancer, chemo-preventive and sunscreen properties	[150,151,152]
6	Eicosane	Antitumour, antifungal activity and bronchodilators	[153,154,155]
7	1-Heptacosanol	Nematicidal, anticancer, antioxidant and antimicrobial properties	[156,157,158]
8	Tetratetracontane	Plant growth production, antioxidant, cytoprotective and anti-inflammatory activities	[113,138,159,160]
9	l-(+)-Ascorbic acid 2,6-dihexadecanoate	Antioxidant food addictive, antimetastatic, anti-invasive, cancer, cardio protective and anti-infertility	[161,162,163]
10	Tridecanoic acid	Antifungal, antibacterial and larvicidal	[164,165,166]
11	Decanedioic acid, dibutyl ester	Antimicrobial, antispasmodic and anti-inflammatory effects	[167]
12	Octadecanoic acid, 2,3-dihydroxypropyl ester	Anticancer, antimicrobial, acidifier, acidulant, arachidonic acid inhibitor and inhibits production of uric acid	[139,141]
13	1,2,3,5-Cyclohexanetetrol	Antioxidant, antimicrobial and anti-inflammatory properties	[168]
14	Phytol, acetate	Anti-inflammatory, antileishmanial, anti-trypanosomal, antimicrobial, anticancer and diuretic	[32,169,170,171]
15	*n*-Nonadecanol-1	Antimicrobial and cytotoxic properties	[172,173]
16	Phytol	Anticancer, antimicrobial, anti-inflammatory, antioxidant activity, diuretic, cosmetics and used in the fragrance industry	[141,174]
17	1,2-15,16-Diepoxyhexadecane	Antitumour and anti-inflammatory properties	[175]
18	1,4-Benzenedicarboxylic acid, bis(2-ethylhexyl) ester	Anticancer properties	[176,177]
19	Vitamin E	Skin repair, enhancing the immune system and has anticancer, antitumour and antioxidant properties	[151,178,179]
20	Flavone	Antibacterial, antimutagenic, antiviral and antioxidant activity	[180,181,182]
21	Campesterol	Anti-inflammatory and anticancer activity	[183,184]
22	Stigmasterol	Anti-inflammatory, anti-asthma, anticancerous, anti-inflammatory, antiarthritic, hypoglycemic, antioxidant and thyroid-inhibiting properties, analgesic, antiosteoarthritic and antimutagenic activity	[141,185,186]
23	Beta-Sitosterol	Reduces cholesterol levels, androgen blocker, anti-amyloid beta and anticancer properties	[141,187]
24	Alpha-Amyrin	Alpha amylase and glucosidase inhibitor, antioxidant, antibacterial and anti-inflammatory properties	[151,188]
25	Simiarenol	Antinociceptive activity	[189]
26	9,12-Octdecadienoic acid (Z,Z)-	Anti-inflammatory, antibacterial, antiarthritic, hepatoprotective, anti-histaminic, anticoronary and anticancer properties	[139,190,191]
27	Dichloroacetic acid, tridec-2-ynyl ester	Cosmetic treatments, anticancer, antimicrobial, antioxidant activity	[192,193]
28	4,4,6a,6b,8a,11,11,14b-Octamethyl-1,4,4a,5,6,6a,6b,7,8,8a,9,10,11,12,12a,14,14a,14b-octadecahydro-2H-picen-3-one	Antibacterial, antioxidant, antitumour and cancer preventives	[194,195]
29	Phenol, 2,4-bis(1,1-dimethylethyl)-	Antibacterial and anti-inflammatory activities	[196]
30	Tributyl acetylcitrate	Anticancer and antimicrobial activities	[197,198,199]
31	9-Octadecenamide	Antimicrobial activity	[148]
32	Alpha. Amyrenone	Antibacterial and antimalarial activities	[200,201]
33	Acetic acid, 3-hydroxy-6-isopropenyl-4,8a-dimethyl-1,2,3,4,5,6,7,8	Antimicrobial activity	[202,203]
34	Stigmasta-3,5-dien-7-one	Anti-diabetic and anticancer properties, free-radical scavenging activity	[204,205,206]
35	Cholest-4-en-3-one	Anti-obesity and an intestinal metabolite of cholesterol	[207,208]

### 2.4. Antibacterial Activity of Leaf and Stem Extracts of B. albostellata

The current interest in herbal plants as therapeutic agents has increased in several parts of the world. This is due to the ever-increasing occurrence of drug-resistant bacteria and the influx of new pathogenic bacterial strains. Active phytochemicals found in hexane, chloroform and methanolic extracts of *B. albostellata* were subjected to antibacterial assays. Various concentrations (3.125, 6.25, 12.25, 25, 50 and 100 mg/mL) were tested against the Gram-positive (*B. subtillus*, methicillin-resistant *S. aureus* and *S. aureus*) and Gram-negative (*E. coli* and *P. aeruginosa*) bacteria. The zone of inhibition of the growth of bacteria was used to evaluate the antibacterial potential of the various extracts. Results presented in Table 11 of certain leaf and stem extracts showed significant inhibition compared to streptomycin and gentamicin (positive controls) (Table 11). The screening was done in triplicate with streptomycin (Gram-positive) and gentamicin (Gram-negative) used as the standard antibacterial positive controls, and 10% DMSO without plant extracts was used as the negative control. Clear zones of inhibition were observed in the leaf and stem crude extracts against the various strains. Significance was established at *p* < 0.05 for all concentrations and treatments. The various extracts and concentrations displayed a variable degree of bacterial growth against various bacterial strains.

As the concentration increased (3.125, 6.25, 12.25, 25, 50 and 100 mg/mL), the zone of inhibition against various bacterial strains also increased. Inhibition against the various bacterial strains for the various extracts was noted at concentrations > 25 mg/mL. The highest inhibitory activity was observed at 100 mg/mL for both leaf and stem extracts for *B. subtillus* and *S. aureus*. MRSA, *E. coli* and *P. aeruginosa* were resistant to the leaf hexane extracts, whilst the stem hexane extracts displayed no inhibition against *E. coli* and *P. aeruginosa* only (Table 11). Gram-positive and Gram–negative bacteria were resistant to all extracts at both 3.125 and 6.25 mg/mL concentrations. Amoo et al. [57] verified the antibacterial activity of *B. albostellata* against *B. subtillis*, *S. aureus* and *E. coli*. However, low activity was observed against Gram-negative bacteria [57]. Matu and Van Staden [209] suggested that a thick murein layer present in the structure of Gram-negative bacteria may prevent the entry of inhibitors. The differences in the bacterial inhibition varied for each crude extract. The leaf methanolic extracts at 100 mg/mL displayed the highest inhibition against all tested bacterial strains. However, the stem methanolic extracts had the highest inhibition against *S. aureus* and *P. aeruginosa* only.

Several notable bioactive compounds found in the leaf and stem extracts of *B. albostellata* using GC-MS analysis were reported to display antibacterial efficiency. The presence of phytol and flavone found in the leaf methanolic extracts of *B. albostellata* could be responsible for the antibacterial effects against the several tested strains. Phytol was reported to severely damage the deoxyribonucleic acid (DNA) of bacteria by inducing oxidative stress [210]. The presence of flavonoids blocks important enzymes that play a significant role in the reproduction, growth, cell rupture or functional modification in bacteria [211]. Stigmasterol, another compound found in the leaf methanolic extract, was reported to act as a lactamase inhibitor, which prevented antibacterial resistance by restoring the vulnerability of the antibiotic resistant bacteria to antibiotics [212]. The mode of action used by most bioactive compounds in treating microbial infections is by interacting with the microbial enzyme system, interfering with nucleic acids, the cell wall and the cell membrane [213,214,215].

Additionally, the antibacterial efficiency in the various extracts may be due to greater solubility of phyto-compounds in polar solvents than non-polar solvents [123]. It was recommended that the inability of plant extracts of other solvent systems to display antibacterial activity against the various bacterial strains could be due to these strains exhibiting some sort of resistance mechanism, e.g., alteration of target sites, enzyme inactivation, reduced drug accumulation or the amount of bioactive compounds present is very low [216]. Extracts of *B. acuminata* [69], *B. cristata* [217], *B. greenii* [57], *B. prionitis* [218] and *B. montana* [125] exhibited antibacterial activity against *B. subtillis* and *S. aureus*. However, *B. cristata* displayed low inhibition against *E. coli* [217], and *B. montana* [125] displayed moderate activity against *E.coli* and *P. aeruginosa*. According to Kumari and Dubey [123], ethanolic leaf extracts of *B. lupulina* inhibited the growth of *E. coli*, *S. aureus* and *P. aeruginosa*, whereas methanolic extracts displayed zones of inhibition against *S. aureus* and no inhibition against *E. coli* and *P. aeruginosa* [219]. Various medicinal plant extracts were reported to display greater activity against Gram-positive bacteria as opposed to Gram-negative bacteria [57,220,221]. The positive results in the present study could be attributed to the location of collected plant material, active constituents found in the different extracts, various extraction preparation, the broad range of treatment concentrations and the variety of bacterial strains. Therefore, the active constituents found in the different extracts of this plant were effective against both Gram-positive and -negative bacteria. 

Qualitative phytochemical screening and GC-MS revealed various biologically active compounds which have been known to contain diverse activities that may help protect against chronic diseases [66,67]. Several compounds found in the extracts of *B. albostellata* were also noted in other species of *Barleria* [124,131,137,138,140,141]. Additionally, the phyto-constituents found in the leaf and stem crude extracts could inhibit the growth of various pathogenic strains. Various medicinal plant extracts were reported to display greater activity against Gram-positive bacteria as opposed to Gram-negative bacteria [57,220,221]. The antibacterial efficiency in the various extracts may be due to greater solubility of phyto-compounds in polar solvents than non-polar solvents [136]. 

## 3. Materials and Methods

### 3.1. Plant Materials

Leaves and stems of *B*. *albostellata* were collected from the University of KwaZulu-Natal, Westville campus (29°49′51.6″ S, 30°55′30″ E), Durban, South Africa. A voucher specimen (7973000) was deposited in the Ward Herbarium of the University of KwaZulu-Natal, Life Sciences, Westville campus. 

### 3.2. Organoleptic Evaluation

The evaluation of crude leaf and stem material was completed with the aid of sensory organs following standard methods [221]. This protocol uses colour, odour, taste and texture. Organoleptic assessment is accomplished using organs of sense and describing specific features of the material. This assessment is regarded as a first step towards establishing the identity and degree of purity of the sample [222].

### 3.3. Preparation of Crude Extract

For the preparation of the crude extract, leaves and stems were oven-dried for 2 weeks at 35°. The dried materials were crushed to a fine powder with the aid of a mechanical blender (Russel Hobbs, model: RHB315). The powdered material underwent sequential extraction using various solvents (hexane, chloroform and methanol) in a Soxhlet apparatus. Approximately 10 g of powdered leaves were placed into a round-bottom flask containing 100 mL of hexane, the appropriate solvent, and boiled for 3 h at 40 °C. The extracted solution was filtered (Whatman^®^ No. 1 filter paper) and retained. This procedure was conducted in replicates. Consecutive extractions of chloroform followed by methanol were achieved. Each solvent extraction followed the same process as mentioned above. Successive extractions were performed on the leaf and stem material.

#### Evaporation and Concentration

The concentration of each extract was left to evaporate in a dark fume-hood, at room temperature. The dried extracts were stored in airtight, labelled glass jars to prevent the material from reacting with the atmospheric humidity. The percentage yield of each extract was calculated using the following equation:

$$\mathrm{Extract}\;\mathrm{Yield}\;(\%)=\frac{\mathrm{Weight}\;\mathrm{of}\;\mathrm{dried}\;\mathrm{extract}\;(g)}{\mathrm{Weight}\;\mathrm{of}\;\mathrm{plant}\;\mathrm{material}\;(g)}\times 100$$

### 3.4. Qualitative Phytochemical Analysis

Preliminary phytochemical screening was carried out on the powdered leaf and stem material and chemically tested for the presence of various constituents using standard protocols [223,224,225,226].

### 3.5. Fluorescence Analysis

Fluorescent analysis of the dried powdered plant material plays an important role in the determining the quality and purity of the tested drug. A small quantity of the dry plant powder (leaves and stems) was placed separately onto clean microscope slides. Two drops of each prepared reagent were dispensed, mixed gently by slanting the slide and allowed to stand for 3 min for the thorough absorption of the solution by the plant powder. The slides were then viewed using a Nikon Eclipse microscope, using bright field light and UV-2A (excitation 320/380) illumination. The colours attained by the application of various reagents were recorded. Fluorescence analysis of the leaf and stem powder was carried out using the standard method [227,228].

### 3.6. Gas Chromatography-Mass Spectrometry (GC-MS)

This analysis is used to examine liquid, gaseous or solid samples and produce several different peaks in the gas chromatogram. Each peak generates a specific mass spectrum which is used for compound identification. Leaf and stem methanolic extracts were analysed using the GC-MS (QP-2010 Ultra SE, Shimadzu, Kyoto, Japan) instrument, with an Rx_5Sil Ms capillary column (0.25 μm internal diameter and 0.25 μm film thickness) from Restek (Bellefonte, PA, USA). The carrier gas, helium, had a flow rate of 0.96 mL/min, a total flow of 4.9 mL/min and a linear velocity of 36.7 cm/sec at a purge flow of 3.0 mL/min. The injection temperature was set at 250 °C. The oven temperature was set at 50 °C and held for 1 min, increased to 310 °C and held for a further 10 min. Chemical compounds (analytes) were identified by relating their retention times with those of the polychlorinated biphenyl (PCB) standards found in the National Institute of Standards and Technology (NIST) library. This analysis was conducted at the Department of Chemistry at the University of KwaZulu-Natal, Westville campus. 

### 3.7. Antibacterial Bioassay 

Crude (hexane, chloroform and methanol) leaf and stem extracts were transferred to Eppendorf centrifuge tubes, dissolved in 10% dimethyl sulfoxide (DMSO) at various concentrations of 100, 50, 25, 12.5, 6.25 and 3.125 mg/mL and homogenised using a vortex. The prepared sample was stored at −4 °C until further use. The prepared crude extracts were subjected to antibacterial assays. Leaf and stem samples were tested against Gram-positive bacteria (*Bacillus subtillus* ATCC 6633, *methicillin*-resistant *Staphylococcus aureus* ATCC 43300 and *Staphylococcus aureus* ATCC 25923) and Gram-negative bacteria (*Pseudomonas aeruginosa* ATCC 25783 and *Escherichia coli* ATCC 35218). These bacterial strains were supplied by Professor Johnson Lin, School of Life Sciences (Microbiology Department), University of KwaZulu-Natal, and maintained in 75% glycerol at −80 °C before the experiment was conducted.

In vitro antibacterial screening of the prepared extracts was conducted using the agar disc diffusion technique as per the Clinical and Laboratory Standards Institute (CLSI) guidelines [229]. Both Gram-positive and Gram-negative bacteria from stock cultures were sub-cultured onto fresh agar plates and incubated overnight at 37 °C. Glass test tubes containing distilled water (15–20 mL) were autoclaved at 121 °C for 1 h. Colonies of bacteria from each Petri plate were harvested with a sterile loop and inoculated by transferring a loopful into glass test tubes containing 15 mL of sterile distilled water (0.5 McFarland scale). The absorbance of each bacterial culture was measured, adjusted and diluted to attain a viable cell count using the Cary 60 UV-Vis spectrophotometer. 

Each bacterial strain was separately smeared uniformly over the surface of the Mueller–Hinton agar plates with a sterile cotton swab. Sterile Whatman filter paper No. 1 discs (diameter 6 mm) were impregnated with 20 μL of the respective extract concentrations (3.125, 6.25, 12.5, 25, 50, 100 mg/mL) and dried at room temperature for 1 h before use [230]. The prepared sterile discs containing extracts were placed carefully onto the agar using sterile forceps. Petri plates were sealed and incubated for 24 h at 37 °C. Zones of inhibition evident around the filter paper were taken as positive results. The diameters of inhibition were measured and photographed within 18–24 h after incubation to determine if the extract exhibited any antibacterial activity. Filter paper discs loaded with streptomycin and gentamycin were used as positive controls and 10% DMSO as the negative control [231]. The analyses were conducted in triplicate, and data were presented as mean ± standard deviation.

### 3.8. Statistical Analysis

All experiments conducted for the antibacterial assay were carried out in triplicate. Values were expressed as mean ± standard deviation (significant at *p* < 0.05 level). Antibacterial data were statistically analysed using the one-way analysis of variance (ANOVA). 

## 4. Conclusions

It is evident from the present study that the qualitative colour tests, fluorescence and GC-MS analysis that the leaves and stems of *B. albostellata* possess biologically active compounds. Important compounds identified in leaf and stem extracts of *B*. *albostellata* were alkaloids, flavonoids and phenols. These compounds are known to display several diverse activities that may help protect against chronic diseases. Major pharmacologically active compounds found in the extracts were alpha-amyrin, flavone, phenol, phytol, phytol acetate, squalene and stigmasterol. Additionally, phyto-constituents found in the hexane, chloroform and methanol leaf and stem extracts of *B*. *albostellata* could inhibit the growth of various pathogenic strains (*p* < 0.05). Other solvents such as ethanol and acetone can be used in extracting phytochemical compounds from the leaves and stems. These extractions can be subjected to antibacterial assays in order to evaluate its potency against various pathogenic strains. Further studies should be conducted on the isolation, identification and characterisation of the bioactive compounds in *B*. *albostellata* that may be responsible for its bioactivity. This is important to further understand the mechanisms involved in the antibacterial activity. The bioactive compounds and pharmacological activities of *B. albostellata* will provide a basic understanding of the importance of this species as a medicinal plant and a potential source for novel and useful drugs. Additionally, other parts of the plant such as the flowers and roots should assessed for their safety and bioactivity and to identify any new therapeutic compounds or drug leads. 

## Figures and Tables

**Table 1:** Organoleptic features of different parts of *B. albostellata*.

Organoleptic Features	Leaf	Stem
Colour	Grey-green on both surfaces but lighter on the lower side	Yellow-buff on uppermost internodes, white or cream below
Odour	Slightly aromatic	Inodorous
Taste	Acrid	Acrid
Texture	Velvety	Woody, glabrescent

**Table 2:** Percentage yield of the leaf and stem crude extracts of *B. albostellata*.

Crude Extract	Leaves	Stem	Leaves	Stem	Leaves	Stem
	Dried Extract Yield (g)		Percentage Yield (%)		Colour	
Hexane	0.139	0.194	1.39	1.94	Dark yellow	Light yellow
Chloroform	0.265	0.219	2.65	2.19	Dark green	Light green
Methanol	1.678	0.938	16.78	9.38	Dark brown	Light yellow

**Table 3:** Preliminary phytochemical screening for major classes of compounds in hexane, chloroform and methanolic leaf and stem extracts of *B*. *albostellata*.

Compound Group	Phytochemical Test	Leaves			Stems		
		Hexane	Chloroform	Methanol	Hexane	Chloroform	Methanol
Alkaloids	Dragendorffs	+++	+++	+++	++	+++	+++
	Mayers	+++	−	++	−	−	+−
	Wagners	+++	+++	+−	+−	+++	+−
Amino acids	Ninhydrin	+−	−	+−	−	−	++
Carbohydrates	Benedicts	−	−	+++	−	++	−
	Fehlings	−	+++	+−	+++	+++	++
	Molisch	−	+−	+−	−	+−	−
Fixed oils and fats	Filter paper	++	+−	+−	−	−	−
Flavonoids	Lead acetate	+++	+++	+−	+++	+++	+−
Mucilage and Gums	Ruthenium	+++	+++	+++	++	+++	+++
Phenols	Ferric trichloride	+−	++	+++	+−	+−	+−
Saponins	Froth	+−	++	+−	+−	+−	++
	Foam	+−	+−	+++	+++	+++	++
Terpenoids	Chloroform	++	++	++	+−	+−	++
Sterols	Salkowski’s	−	++	+−	−	−	+−

Intensity of reaction: (−) no observed changes, (+−) low intensity, (++) medium intensity, (+++) high intensity.

**Table 4:** Phytochemical compounds identified in leaf hexane extracts of *B. albostellata* by GC-MS analysis.

Peak	Retention Time	Phytochemical Compound	Molecular Formula	Molecular Weight	CAS No.	Area %
1	17.738	Pentadecanoic acid	C_15_H_30_O_2_	242	1002-84-2	1.02
2	19.422	9,12,15-Octadecatrienoic acid, (Z)	C_18_H_30_O_2_	278	463-40-1	1.25
3	19.628	Octadecanoic acid	C_18_H_36_O_2_	284	57-11-4	1.09
4	24.591	13-Docosenamide, (Z)	C_22_H_43_NO	337	112-84-5	1.12
5	24.804	Squalene	C_30_H_50_	410	111-02-4	1.06
6	26.742	Eicosane	C_20_H_42_	282	112-95-8	1.39
7	26.784	1-Heptacosanol	C_27_H_56_O	396	2004-39-9	1.21
8	28.136	Tetratetracontane	C_44_H_90_	618	7098-22-8	3.25
9	29.965	l-(+)-Ascorbic acid 2,6-dihexadecanoate	C_38_H_68_O_8_	652	28474-90-0	2.85

**Table 5:** Phytochemical compounds identified in leaf chloroform extracts of *B. albostellata* by GC-MS analysis.

Peak	Retention Time	Phytochemical Compound	Molecular Formula	Molecular Weight	CAS No.	Area %
1	17.738	Pentadecanoic acid	C_15_H_30_O_2_	242	1002-84-2	1.02
2	19.418	9,12,15-Octadecatrienoic acid, (Z)	C_18_H_30_O_2_	278	463-40-1	1.04
3.	19.646	Tridecanoic acid	C_13_H_26_O_2_	214	638-53-9	1.01
4	19.674	Decanedioic acid, dibutyl ester	C_18_H_34_O	314	109-43-3	1.02
5	24.168	Octadecanoic acid, 2,3-dihydroxypropyl ester	C_21_H_42_O	443	123-94-4	1.00
6	24.605	13-Docosenamide, (Z)	C_22_H_43_NO	338	112-84-5	1.03
7	28.120	Tetratetracontane	C_44_H_90_	619	7098-22-8	1.00

**Table 6:** Phytochemical compounds identified in leaf methanol extracts of *B. albostellata* by GC-MS analysis.

Peak	Retention Time	Phytochemical Compound	Molecular Formula	Molecular Weight (g/mol)	CAS No.	Area %
1	14.743	1,2,3,5-Cyclohexanetetrol	C_6_H_12_O_4_	619	53585-08-3	3.63
2	17.057	Phytol, acetate	C_22_H_42_O	339	0-00-0	7.29
3	19.514	*n*-Nonadecanol-1	C_19_H_40_O	285	1454-84-8	5.35
4	19.747	Phytol	C_20_H_40_O	297	150-86-7	4.66
5	21.140	1,2-15,16-Diepoxyhexadecane	C_16_H_30_O_2_	254	0-00-0	3.10
6	24.591	13-Docosenamide, (Z)	C_22_H_43_NO	337	112-84-5	2.46
7	24.897	1,4-Benzenedicarboxylic acid, bis(2-ethylhexyl) ester	C_24_H_38_O_4_	391	6422-86-2	6.46
8	25.388	Squalene	C_30_H_5_O	410	111-02-4	5.39
9	27.340	1-Heptacosanol	C_27_H_56_O	397	2004-39-9	4.27
10	27.579	Vitamin E	C_29_H_50_O_2_	431	59-02-9	3.67
11	27.723	Flavone, 4′,5-dihydroxy-6,7-dimethoxy-	C_17_H_14_O_6_	314	6601-62-3	11.69
12	28.491	Campesterol	C_28_H_48_O	401	474-62-4	5.16
13	28.705	Stigmasterol	C_29_H_48_O	413	83-48-7	4.01
14	29.257	Beta-Sitosterol	C_29_H_50_O	415	83-46-5	6.70
15	29.763	Alpha-Amyrin	C_30_H_50_O	427	638-95-9	3.22
16	30.390	Simiarenol	C_30_H_50_O	427	1615-94-7	4.25

**Table 7:** Phytochemical compounds identified in stem hexane extracts of *B. albostellata* by GC-MS analysis.

Peak	Retention Time	Phytochemical Compound	Molecular Formula	Molecular Weight	CAS No.	Area %
1	17.759	Pentadecanoic acid	C_15_H_30_O_2_	242	1002-84-2	1.00
2	19.373	9,12-Octdecadienoic acid (Z)	C_18_H_32_O_2_	280	60-33-3	2.33
3	19.433	Dichloroacetic acid, tridec-2-ynyl ester	C_15_H_24_C_l2_O_2_	306	0-00-0	1.52
4	19.646	Octadecanoic acid	C_18_H_36_O_2_	284	57-11-4	1.52
5	19.673	Decanedioic acid, dibutyl ester	C_18_H_34_O_4_	314	109-43-3	1.39
6	24.601	13-Docosenamide, (Z)	C_22_H_43_NO	337	112-84-5	1.32
7	28.125	Tetratetracontane	C_44_H_90_	618	7098-22-8	1.45
8	28.796	4,4,6a,6b,8a,11,11,14b-Octamethyl-1,4,4a,5,6,6a,6b,7,8,8a,9,10,11,12,12a,14,14a,14b-octadecahydro-2H-picen-3-one	C_30_H_48_O	424	0-00-0	2.35

**Table 8:** Phytochemical compounds identified in stem chloroform extracts of *B. albostellata* by GC-MS analysis.

Peak	Retention Time	Phytochemical Compound	Molecular Formula	Molecular Weight	CAS No.	Area %
1	12.711	Phenol, 2,4-bis(1,1-dimethylethyl)-	C_14_H_22_O	206	96-76-4	1.20
2	17.750	Pentadecanoic acid	C_15_H_30_O_2_	242	1002-84-2	1.00
3	19.641	Octadecanoic acid	C_18_H_36_O_2_	284	57-11-4	1.06
4	19.675	Decanedioic acid, dibutyl ester	C_18_H_34_O_4_	314	109-43-3	1.07
5	24.168	Octadecanoic acid, 2,3-dihydroxypropyl ester	C_21_H_42_O_4_	358	123-94-4	1.03
6	24.612	13-Docosenamide, (Z)	C_22_H_43_NO	337	112-84-5	1.21

**Table 9:** Phytochemical compounds identified in stem methanol extracts of *B. albostellata* by GC-MS analysis.

Peak	Retention Time	Phytochemical Compound	Molecular Formula	Molecular Weight	CAS No.	Area %
1	19.514	*n*-Nonadecanol-1	C_19_H_40_O	285	1454-84-8	5.84
2	20.942	Tributyl acetylcitrate	C_20_H_34_O_8_	402	77-90-7	1.05
3	21.139	1,2-15,16-Diepoxyhexadecane	C_16_H_30_O_2_	254	0-00-0	1.23
4	21.967	9-Octadecenamide	C_18_H_35_NO	281	301-02-0	2.19
5	24.588	13-Docosenamide, (Z)	C_22_H_43_NO	337	112-84-5	1.14
6	24.901	1,4-Benzenedicarboxylic acid, bis(2-ethylhexyl) ester	C_24_H_38_O_4_	391	6422-86-2	25.68
7	25.386	Squalene	C_30_H_50_	410	111-02-4	1.39
8	27.337	1-Heptacosanol	C_27_H_56_O	397	2004-39-9	1.72
9	28.703	Stigmasterol	C_29_H_48_O	413	83-48-7	1.89
10	29.252	Beta-Sitosterol	C_29_H_50_O	415	83-46-5	5.68
11	29.453	Alpha. Amyrenone	C_30_H_48_O	425	0-00-0	2.18
12	29.755	Alpha-Amyrin	C_30_H_50_O	427	638-95-9	3.16
13	29.928	Acetic acid, 3-hydroxy-6-isopropenyl-4,8a-dimethyl-1,2,3,4,5,6,7,8	C_17_H_26_O_3_	278	0-00-0	2.01
14	30.195	Stigmasta-3,5-dien-7-one	C_29_H_46_O	411	2034-72-2	1.51
15	30.604	Cholest-4-en-3-one	C_27_H_44_O	385	601-57-0	1.13

**Table 11:** Antibacterial activity of crude extracts from leaves and stem of *B. albostellata* against human pathogenic strains (zone of inhibition, mm).

Strain	Concentration (mg/mL)	Extracts						Positive Control (mg/mL)	
		Leaf Hexane	Leaf Chloroform	Leaf Methanol	Stem Hexane	Stem Chloroform	Stem Methanol	Leaf	Stem
*B. subtillus* (ATCC 6633)	3.125	R	R	R	R	R	R	9.00 ± 1.00	11.00 ± 1.00
	6.25	R	R	R	R	R	R		
	12.5	R	R	R	R	R	R		
	25	R	8.33 ± 1.53	8.00 ± 1.00	R	7.33 ± 0.58	8.00 ± 0.00		
	50	7.67 ± 2.08	7.67 ± 0.58	9.33 ± 0.58	7.67 ± 2.08	8.00 ± 1.00	8.67 ± 0.58		
	100	9.00 ± 3.46	7.00 ± 0.00	10.00 ± 2.00	8.67 ± 1.52	10.00 ± 3.61	9.67 ± 0.58		
Methicillin-resistant *S. aureus* (ATCC 43300)	3.125	R	R	R	R	R	R	9.33 ± 0.58	9.00 ± 1.00
	6.25	R	R	R	R	R	R		
	12.5	R	R	R	R	R	R		
	25	R	8.67 ± 0.58	R	R	R	R		
	50	R	9.00 ± 0.00	8.67 ± 2.08	R	7.33 ± 0.58	8.00 ± 1.00		
	100	R	10.00 ± 0.00	11.00 ± 2.65	10.67 ± 2.31	8.00 ± 1.00	9.00 ± 2.00		
*S. aureus* (ATCC 25923)	3.125	R	R	R	R	R	R	9.67 ± 0.58	10.00 ± 1.00
	6.25	R	R	R	R	R	R		
	12.5	R	R	7.33 ± 0.58	R	R	7.00 ± 0.00		
	25	R	7.67 ± 0.58	8.00 ± 0.00	8.00 ± 1.00	7.33 ± 0.58	8.00 ± 1.00		
	50	7.33 ± 0.58	8.67 ± 0.58	8.67 ± 1.53	9.00 ± 0.00	8.33 ± 0.58	10.00 ± 1.73		
	100	9.33 ± 0.58	9.33 ± 0.58	10.33 ± 1.53	10.33 ± 1.53	9.00 ± 1.73	11.00 ± 2.65		
*E. coli* (ATCC 35218)	3.125	R	R	R	R	R	R	8.67 ± 0.58	9.33 ± 0.58
	6.25	R	R	R	R	R	R		
	12.5	R	R	R	R	R	R		
	25	R	9.67 ± 0.58	R	R	9.33 ± 0.58	R		
	50	R	10.67 ± 1.15	9.00 ± 3.46	R	10.00 ± 1.00	9.67 ± 2.08		
	100	R	12.33 ± 2.08	12.67 ± 0.58	R	11.33 ± 1.15	11.33 ± 1.15		
*P. aeruginosa* (ATCC 25783)	3.125	R	R	R	R	R	R	9.33 ± 0.58	8.67 ± 1.15
	6.25	R	R	R	R	R	R		
	12.5	R	R	R	R	R	R		
	25	R	8.67 ± 1.53	7.33 ± 0.58	R	7.00 ± 0.00	9.33 ± 1.15		
	50	R	9.00 ± 0.00	8.67 ± 1.52	R	8.67 ± 0.58	10.67 ± 2.87		
	100	R	10.00 ± 3.00	14.33 ± 1.53	R	9.67 ± 1.15	12.33 ± 0.58		

R-resistant, positive controls (streptomycin 10 mg/mL (Gram-positive bacteria), gentamicin 10 mg/mL (Gram-negative bacteria)).

## Data Availability

Not applicable.
